# A multi-suckling system combined with an enriched housing environment during the growing period promotes resilience to various challenges in pigs

**DOI:** 10.1038/s41598-022-10745-4

**Published:** 2022-04-26

**Authors:** S. P. Parois, L. E. Van Der Zande, E. F. Knol, B. Kemp, T. B. Rodenburg, J. E. Bolhuis

**Affiliations:** 1 Adaptation Physiology Group, Wageningen University and Research, Wageningen, The Netherlands; 2 PEGASE, INRAE, AGROCAMPUS OUEST, Saint Gilles, France; 3 Topigs Norsvin Research Center, Beuningen, The Netherlands; 4 Animals in Science and Society, Faculty of Veterinary Medicine, Utrecht University, Utrecht, The Netherlands

**Keywords:** Zoology, Animal behaviour, Animal physiology

## Abstract

Little is known about the impact of social and environmental enrichment on improving livestock resilience, i.e. the ability to quickly recover from perturbations. We evaluated the effect of an alternative housing system (AHS) on resilience of pigs, as compared to conventional housing (CONV). The AHS consisted of multi-litter housing during lactation, delayed weaning, extra space allowance and environmental enrichment at all times. We assessed recovery to a 2 h-transport challenge, an LPS injection, 2 h-heat stress and a biopsy wound in 96 pigs. Additionally, indicators of long-term “wear and tear” on the body were determined. AHS pigs had better physiological recoveries with quicker returns to baseline in the transport and LPS challenges, showed lower cortisol accumulation in hairs and lower variance in weight gain over the experimental period compared to conventionally-housed (CONV) pigs. They also had higher levels of natural antibodies binding KLH than CONV pigs. Their response to heat stress revealed a different strategy compared to CONV pigs. Taken together, AHS pigs appear to be more resilient and experience less chronic stress. Enhancing welfare by provision of social and environmental enrichment that better meets the behavioural needs of pigs seems to be a promising approach to improve their resilience.

## Introduction

In current husbandry systems, most pigs seem to cope with the metabolic demands of rapid, efficient growth combined with the multiple acute and chronic stressors they are exposed to such as vaccination, pathogenic load, regrouping, and suboptimal housing conditions. The burden of cumulative stress, however, may cause “wear and tear” on the animals and reduce their resilience, i.e. their capacity to recover from challenges. Resilience can be defined as the capacity of an animal to be minimally affected by a disturbance or to rapidly return to its normal state^1^. As poor resilience may manifest itself as an increased risk to develop behavioural and health problems^2,3^, optimizing resilience is important for the performance and welfare of farm animals^1,4,5^. Studies on farm animals have mainly focussed on enhancing resilience through genetic selection^1,6^, while limited research has been conducted on the effect of the environment in which the animals are reared and kept.

Enrichment is defined as a modification in the environment that improves the biological functioning of animals^7^. Numerous studies have shown that social and/or environmental enrichment promotes natural behaviour^8–13^ and the development of appropriate social skills^14–18^, reduces damaging behaviour^19–21^, leads to a more optimistic emotional state^22^, and reduces stress at weaning^21,23–26^. Less is known, however, on the impact of enrichment on resilience in pigs. It has been suggested, however, that raising animals in housing conditions that promote the satisfaction of their essential behavioural needs may improve resilience^1,13,27–29^.

Recent studies suggest that loss of resilience is characterized by a slow recovery after exposure to a challenge^3^. The recovery rate could thus serve as a putative indicator of (the loss of) resilience. Animals in a state of poor resilience may need only a small disturbance to collapse into a health or behavioural crisis^30–32^. This seems to be the case in, for instance, the occurrence of tail biting in pigs where only a seemingly small change in e.g. climatic conditions or feed can result in a severe outbreak of this injurious behaviour. It has been hypothesized that moving towards the threshold of change from one (normal) into the other (problematic) state, also referred to as a ‘tipping point’, can also be predicted by changes in the dynamics of patterns of generic parameters^30,33^. In pigs, these could, for instance, be weight gain or activity patterns. In line with this, some variables reflecting changes in activity patterns following a porcine reproductive and respiratory virus infection were related to morbidity and mortality of pigs^34^. However, the dynamic indicators mentioned are not pertinent for determining resilience in all cases. The use of more common indicators of recovery from challenge measurable in blood, saliva or as behavioural changes might be useful, as animals develop a combination of physiological and behavioural responses when they encounter perturbations to restore homeostasis^35^. Signs of improved resilience might then be explored through multiple and diverse indicators of recovery from challenges.

A challenge can be defined as an episodic situation where animals may experience acute stress after their external or internal environments changed abruptly^1^. A challenge is characterized by the duration of stimuli, the frequency of their occurrence and the predictability of the onset by the individual. Every response to a challenge has distinct features like response peak, duration, onset and recovery^1^.

In the current study, resilience of pigs kept in an alternative system was compared to that of pigs kept under conventional commercial conditions. The alternative system profoundly differs from conventional conditions. It consists of multi-litter housing during lactation, allowing piglets to mingle with multiple litters during the socialization period, delayed weaning, extra space allowance and provision of rooting substrates. The alternative system resembles the natural situation of pigs more and therefore better meets the behavioural needs of pigs. It was hypothesized that this difference in social and environmental conditions that support the execution of important natural behaviours will result in improved general resilience, i.e. an overall ability to deal with all kinds of unpredictable challenges^36^. Resilience was probed in-depth by measuring the speed of recovery from a range of (mild) challenges. The series of perturbations selected were known to be challenging for pigs, and similar to events that often occur, either successively or simultaneously, in commercial pig husbandry conditions. Chronic indicators of stress, for instance cortisol in hairs, were also measured to capture “wear and tear” on the body, as well as fluctuations in weight development. To assess a full picture of the general resilience of pigs, challenges varied in nature, strength and duration to explore each aspect of recovery.

## Material and methods

Established principles of laboratory animal use and care and the Dutch law on animal experiments were followed. They comply with the European Directive 2010/63/EU on the protection of animals used for scientific purposes. The Animal Care and Use Committee of Wageningen University approved the experiment (AVD1040020186245). All methods applied in the study were performed in accordance with the ARRIVE guidelines and regulations. Due to the difference in housing system according to the treatment group, the investigators were aware of the treatment of the animals when collecting samples. However, lab analyses of tear staining measurements on pictures, behavioural observations during the transport challenge, respiratory rate and metabolic chamber parameters during the heat stress challenge, wound healing areas on pictures and scores related to organs during dissections were done blindly. The sample size estimation was based on the rise in cortisol levels using results obtained in pigs subjected to transport stress^36^ (α = 5%, power = 80%, SD = 2.42, δ = 2.8).

### Animals

A total of 144 Tempo × Topigs-20 pigs (n = 71 females; n = 73 males) were used during the experiment, spread over three batches (n = 48 pigs per batch). Piglets were offspring from 24 multiparous sows. During lactation, half of the sows and their piglets were housed in a conventional farrowing pen (CONV) (mean ± SD; sow parity = 4.2 ± 1.8) and the other half in an alternative group housing system (AHS) (parity = 4.0 ± 1.7) at the Swine Innovation Centre (Sterksel, The Netherlands). The piglets were not castrated, nor were their tails docked or teeth clipped. Average birth weight was similar for piglets from both systems: 1.46 ± 0.28 kg for the CONV and 1.44 ± 0.27 kg for the AHS.

### Housing systems

#### From birth to 9 weeks of age

Piglets were raised in two different housing systems (similar to van Nieuwamerongen et al*.*^21^). The AHS comprised of five farrowing pens of 3.2 × 2.2 m (mix of solid 2.2 × 2.2 m and slatted floor 1.0 × 2.2 m), adjacent to a communal area of 11.1 × 2.80 m (solid floor). Next to the communal area, were a dunging (2.8 × 3.3 m, slatted floor) and a feeding area (4.2 × 3.3 m, solid floor). Enrichment was provided in the form of four jute bags (110 × 80 cm) and during the first week a slice of straw was added to the farrowing pens (approximately 2.5 kg per pen). One week before the expected farrowing date, five sows per batch were put in this system. Two days before the expected day of farrowing, sows were moved to a farrowing pen and confined in a farrowing crate (1.9 × 0.6 m). Two days after farrowing, they were allowed to access the full system again. Newly born piglets were kept with their own litter in the farrowing pens for 1 week, after which they could access the entire system and mingle with the other litters. Piglets were provided with a heated piglet nest next to the farrowing pens (0.7 × 1.6 m), with a temperature of 33–35 °C (day 1 till day 7), 29–31 °C (day 7 till day 25) and 23–26 °C (day 25 till weaning). Piglets were fed in round bowls until 5 weeks of age and from a sensor-controlled automatic feeder (Rondomat, from 3 weeks of age). Besides this, the piglets could participate in feeding with the sows, which were fed in a large trough placed on the floor. Ingestion of solid feed was stimulated with the use of intermittent suckling from week 5 of age onwards. AHS piglets were weaned at an average of 62.6 ± 1.9 days and a body weight of 26.6 ± 4.9 kg. They received a starter diet from 35 days onwards.

In the CONV system, piglets were kept in farrowing pens of 2.8 × 1.8 m until weaning. Sows were confined in a crate (1.9 × 0.6 m). The floor consisted of metal slats within the crate. There was a solid floor of 1.2 × 0.3 m with heating lamp for the piglets and the remaining area consisted of plastic slats. Piglets received additional creep feed in the farrowing pens from 1 week after birth. CONV piglets were weaned at 27.4 ± 1.2 days of age and 8.7 ± 1.3 kg. After weaning, CONV piglets were housed with their litter mates in nursery pens of 3.18 × 1.0 m (0.40 m^2^ per piglet) for five additional weeks with a chain and a jute bag as enrichment. They received a commercial weaner diet for 10 days after weaning and a starter diet, similar to that provided to AHS piglets, from 35 days onwards.

Lights were on from 07:00 h till 19:00 h in both systems, giving the sows and piglets a 12 h light regime with 115 Lux. Besides that, the AHS had natural daylight through two windows. Transition between day and night light settings was done progressively in 10 min. Ambient temperature was 23 °C in both systems. Water was available ad libitum in both systems.

#### From 9 weeks of age onwards

After weaning of the AHS piglets at approximately 9 weeks of age, all piglets were moved to the Carus research facilities in Wageningen, the Netherlands. They were mixed in groups of six unfamiliar piglets originating from the same system. Litter, sex and weight were balanced between pens. Piglets were selected based on their sex (50:50% male and female), and weight at birth in order to choose piglets representative of the full litter. Per litter, 6 piglets were selected: 2 piglets with a birth weight between the minimum weight of the litter + 10% and the 1st weight quartile (light); 2 piglets with a birth weight between the 1st and 3rd quartile (medium); 2 piglets with a birth weight between the 3rd quartile and the maximum weight − 10% (heavy).

AHS pigs were housed in a 2.40 × 4.67 m pen, i.e. double the size of a conventional pen (1.87 m^2^ per pig), enriched with deep straw, peat and sawdust bedding, which was replenished regularly (2.5 kg of straw and 30 L of sawdust every day, 22.5 L peat every week). Besides that, AHS pigs were provided with a handful of hay, alfalfa or cardboard egg trays once a week and a chain, jute bag or rope (rotation every week). They were also provided with one extra toy (either a biting ball on a chain, a free chewing ball for dogs, a tyre dog toy, a porcichew^®^ toy, a green MS Schippers Bite cylinder^®^, or a green MS Schippers Cross^®^) which was changed every 2 days to preserve the attractiveness of the toy. CONV pigs were housed in standard pens of 1.20 × 4.67 m with conventional space allowance (0.93 m^2^ per pig), with a partly solid and partly slatted floor without substrate. CONV piglets were provided with a ball and a chain with screws, which were not changed. AHS and CONV pens were placed alternately in the rooms. Pigs were all fed the same feed (a standard commercial diet for growing pigs) ad libitum from a single pig feeder and water was available ad libitum.

The light regime was similar to that before 9 weeks of age, giving the pigs 115 Lux in the pens during the day (from 7:00 h to 19:00 h; 5000 K ultraviolet A at an intensity of 42, 2700 K at 60) and 30 Lux during the night (5000 K ultraviolet A at an intensity of 3, 2700 K at 0). The transition between the day and night rhythm was done progressively for 10 min. No natural day light was available. Temperature was kept at 23 °C for the first 2 days, then at 22 °C for the 2 subsequent days and at 21 °C onwards.

### Challenges

**Figure 1:**
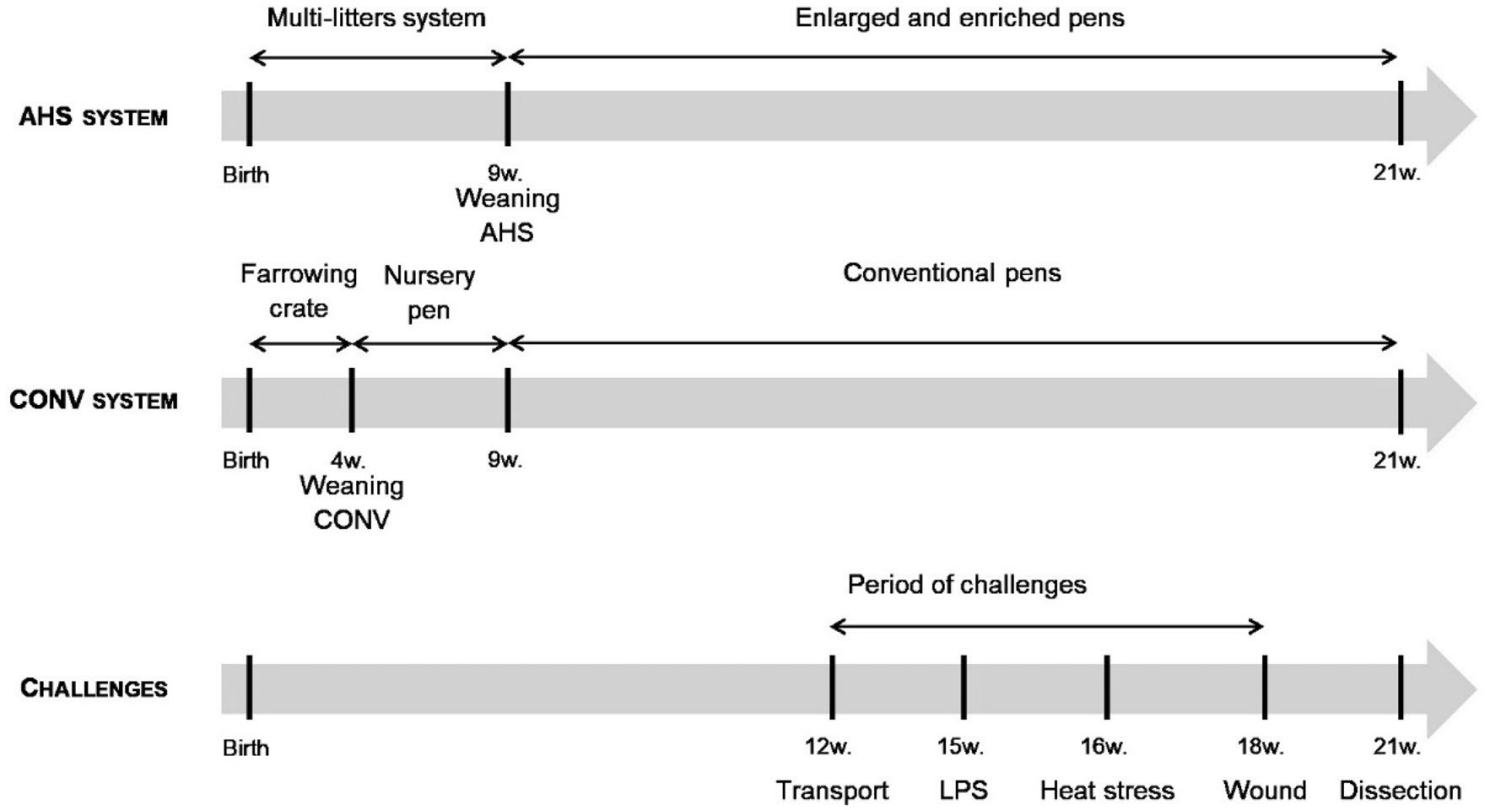
Schematic view of the experiment from birth till dissection of pigs housed in an alternative (AHS) or conventional (CONV) system.

Resilience of the pigs in the two housing systems was assessed by following the recovery process of the animals after submission to different successive challenges in the following order: a 21 h-isolation challenge (not detailed in this paper), a 2 h-transport challenge, an LPS injection to induce a sickness response, a 2 h-heat stress challenge and a wound. Figure 1 summarizes the order and time of the challenges described in this paper. Order of exposing pigs to the challenges was always balanced for housing system. Four animals per pen (Focals, two males and two females) were exposed to the experimental challenges, while two other pigs served as companions (i.e. the two pigs who most deviated from the average pen weight).

#### Transport challenge

At the age of 83.0 ± 1.7 days (weight: 41.6 ± 5.5 kg), 94 focal pigs experienced a 2 h transport. At this point, one of the 96 focal pigs was a tail biter (CONV housing). It was euthanized using an injection of pentobarbital (Euthasol^®^ Vet) and replaced by a companion pig after the transport challenge. Neither of those two pigs were exposed to the transport challenge. Pigs were loaded in a trailer with a thin layer of sawdust on the floor and were transported from 8:00 to 10:00 h. All pigs from one batch (from both housing systems) were mixed in the same trailer, which created both a metabolic and social challenge. To follow the recovery of the animals, blood samples were drawn 24 h before the transport challenge (baseline), when they came back from the 2 h-transport (referred to as 0 h), and at 3 h, 24 h and 48 h after the end of transport.

The day before the transportation, pigs were marked with paint to individually track their behaviour in the trailer during the 2 h-transport challenge. Number of aggressive acts (either a push, a bite, a knock, a mount or a fight) and lying behaviour were continuously scored from video by a single trained observer using the software Observer 14.2 (Noldus Information Technology, Wageningen, The Netherlands). The percentage of time spent lying was averaged over 30-min intervals. The total number of aggressive acts and the total number of posture changes were summed over the 2 h period. Because of poor lighting in the trailer causing difficulties to observe the pigs before sunrise, the first half-hour of batch 1 was excluded from analysis.

#### LPS challenge

At the age of 104.4 ± 1.7 days (weight: 60.0 ± 7.5 kg), 92 focal pigs were injected in the ear vein with 2 μg of LPS/kg of body weight (LPS sigma L4391 *Escherichia coli* O111 B4). Four focal pigs were not subjected to the challenge as they were sick and on antibiotic treatment at the day of challenge. For time constraints, the pigs were challenged in two different groups on two consecutive days, balanced for housing system. Pigs were injected in their home pen in one of their ears. Blood samples were collected at 24 h before (baseline) and 1 h, 3 h, 5 h and 24 h after the injection. At each of these time points, rectal temperature was measured before taking a blood sample. Pigs were habituated to rectal temperature measurements in the weeks prior to the challenge to prevent a temperature change related to handling stress.

#### Heat stress challenge

At the age of 111.0 ± 1.9 days (weight: 68.3 ± 6.9 kg), the 96 focal pigs were group-wise subjected to a heat stress challenge. Due to time and space constraints, the pigs were tested on two different days. The focal animals were moved per pen to climatic respiration chambers (4.5 m × 2.5 m). This was done the day before the challenge at 13:00 h to habituate them to the new environment. The focal animals were housed in pens of 3.5 m × 1.8 m (1.6 m^2^ per pig) with a partly solid and partly slatted floor. Feed and water were available ad libitum. A ball on a chain was provided as a toy. The light schedule was similar to that of the regular room. The ambient temperature was set at 21 °C. During the heat challenge, the temperature raised in 2 h (from 8:30 to 10:30 h) from 21 to 35 °C, stayed at 35 °C for two extra hours and returned to 21 °C in the following 2 h. Humidity was set at 50%. The following day, at 8:00 h, the pigs were moved back to their original room.

From 13:00 h on the day before heat stress until 8:00 h on the day of the heat stress challenge, heat production (kJ/kg^0.75^/day), activity (counts), O_2_ consumption (L/kg^0.75^/day), CO_2_ production (L/kg^0.75^/day) and CH_4_ production (L/climate respiration chamber/day) were measured every 5 min as described before^37^. Average values per relevant time period (baseline during the day, baseline during the night from 20:00 to 8:00 h (based on 2 days, the day before and the day after the challenge), during the 2 h temperature increase, during the 2 h period of temperature at 35 °C, and during the 2 h temperature decrease were calculated. At the individual level, the respiration rate per min was measured by counting belly movements of each animal for a period of 30 s and by doubling the amount obtained. Respiratory rates were measured three times on the day before the challenge at 12:00 h in their home pen; at 15:00 h and 16:00 h in the climatic chambers; the day of the heat challenge every 30 min between 8:00 and 15:00 h and at 16:00 h; the day after the challenge at 8:00 h in the climatic chambers before leaving, and at 12:00 h in their home pen. Feed intake was determined for a period of 19 h from entering until leaving the respiratory chambers.

#### Wound healing challenge

At the age of 123.0 ± 1.7 days (weight: 88.5 ± 13.3 kg), the 96 focal pigs were subjected to a fat biopsy to study their wound healing. A wound of a diameter of 0.7 cm and a length of 3.5 cm was created in the fat from the neck by a penetrating gun as described by Baes et al.^38^. Briefly, the pigs were restrained with a nose sling during the procedure. The location sampled was cleaned with betadine soap before the biopsy. Between each pig, the needle of the gun was cleaned with a solution of 70% ethanol. After the biopsy was done, the wound was sprayed with betadine to prevent infection.

The wound healing process was followed by measuring the perimeter of the wound from photographs taken, without restraining the pigs, by a digital camera daily for 10 days and on day 15 after the biopsy. If needed, the wound was cleaned with water before taking a picture. Measurements were made by a single trained person, blind to treatment, using the ImageJ software^39^ to delimit the wound perimeter. The length of a sticker placed close to the wound was used as a scale to standardize the measurements.

### Indicators measured during the challenges

#### Weights

All pigs were weighed 24 h before and 24 h after the start of each challenge. Relative weight change was estimated as follows: $\frac{\left(Final weight-Initial weight\right)}{Initial weight}$.

#### Blood samples

The animals were restrained with a nose sling during the blood sampling procedures. Blood samples were collected from the jugular vein. The blood (10 ml) was distributed as follows: 4 ml in EDTA tubes, 4 ml in heparin tubes and 2 ml in serum tubes. The EDTA and heparin tubes were centrifuged at 1500*g* for 10 min at + 4 °C. The serum samples were kept at ambient temperature for 30 min and thereafter were centrifuged at 1500*g* for 10 min at 4 °C. Plasma and serum were stored at − 20 °C until laboratory analyses.

Cortisol assays were performed in EDTA samples using the cortisol RIA kit from Immunotech (Beckman-Coulter, ref IM1841, Czech Republic). Glucose concentrations were measured in EDTA samples using a GOD-PAP kit (Hoffmann-La Roche, Switzerland). Non-Esterified Fatty Acids (NEFA) concentrations were measured in serum samples using a NEFA kit (WAKO Chemicals GmbH, Germany). Haptoglobin levels in heparin plasma were measured using the kit PHASE TM Haptoglobin Assay from Tridelta Development Limited (ref TP-801, Ireland). Titers of IgG and IgM in heparin samples binding keyhole limpet hemocyanin (KLH), myelin basic protein (MBP) and phosphoryl choline-conjugated to Bovine Serum Albumin (PC-BSA) were measured as described previously^40^. For IgG and IgM determination, only the sampling points 24 h pre-challenge, 24 h post-challenge and 48 h post-challenge were used. Urea concentrations were measured in EDTA samples using the enzymatic colorimetric test (ref 10506, HUMAN Gesellschaft für Biochemica und Diagnostica mbH, Wiesbaden, Germany).

#### Hair samples

The animals were shaved at 11 weeks of age before the start of the period with challenges, and at 18 weeks of age simultaneously with the biopsy, to determine the accumulation of cortisol over this period. The shaving area of about 225 cm^2^ was located at the same spot for all piglets to avoid potential bias related to the body part. The location was close to the hip of the animals, at the connection between the abdominal area and the hind leg. A one-use surgical razor was used for each animal. The experimenter wore gloves to avoid contamination of the samples. At 11 weeks, only the right side of the animals was shaved, while two sides were shaved at 18 weeks. This enabled to distinguish the effect of the period of challenges between 11 and 18 weeks (regrowth of the right side between the two shaving points) from the full life of the animals from birth onwards (left side never shaved). Samples were stored in aluminium foils at room temperature in the dark until analysis.

Hairs were washed three times: once in PBS and twice in isopropanol to remove any dirt and dust and were dried for 96 h at 37 °C. To extract the cortisol from the hairs, the samples were first cut with scissors in small pieces before being grinded with a tissue lyser. The cortisol from the hair powder was then extracted with methanol for 24 h. The extract was dried in a speedvac for 3 h and dissolved in a phosphate buffer. Cortisol concentration was determined using the high sensitivity salivary cortisol ELISA kit (ref 1-3002) from Salimetrics (Pennsylvania, USA).

#### Skin lesions

Skin lesions were scored 24 h before and 24 h after the start of each challenge according to the Welfare Quality Assessment Protocol^®^ for pigs^41^, except for the wound healing challenge, when it was done simultaneously to the biopsy. Briefly, the body of the pig was divided into 5 separate regions: 1—ears, 2—front (head to the back of the shoulder), 3—middle (back of the shoulder to the hind quarters, 4—hind quarters, and 5—legs (from the accessory digit upwards). A scratch longer than 2 cm was considered as one lesion and two parallel scratches with up to 0.5 cm space in between were considered to be one lesion. Wounds smaller than 2 cm were considered as one lesion. Bleeding wounds, healed wounds of more than 5 cm as well as deep and open wounds of more than 5 cm were never observed. The number of lesions from the different body regions were summed to create a global score.

#### Tear staining

Photographs of the left eye of each focal pig were taken 8 times during the experiment to measure tear-staining. This was done 24 h before and 24 h after transport, LPS and heat stress challenges, right before the biopsy and before slaughter. Measurements were made on photographs by a single experienced person, blind to treatment, using the ImageJ software^39^ to delimit the tear perimeter. The length of the iris was used as a scale to standardize the measurements. All the brownish areas on the direct periphery of the eye (bottom of the upper eyelid, top of the lower eyelid, internal and external corners) were recorded^42^. The variable analysed was the cumulative area covered by the stain.

#### Pathological examination at slaughter

At the age of 140.0 ± 1.9 days (weight: 95.2 ± 8.5 kg), the 96 focal pigs were exsanguinated after an electrical stunning for dissection purpose. The judging of pathological changes was done blind to the housing treatment of the pigs. The heart was examined for pericarditis, the lungs for pneumonia and pleurisy, and the stomach for lesions and ulcers. The heart of the pigs was assessed for pericarditis with a score of 0 (no pericarditis) to 3 (severe pericarditis)^43^. Lung lesions for pneumonia were scored with a scale ranging from 0 (no lesion) to 28 (total pulmonary consolidation)^44^. Pleurisy was scored for the entire lungs ranging from 0 (no adhesion between the lobes) to 4 (lungs fully adherent to the thoracic cavity). Stomach wall damage at the pars oesophagus was assessed with a score of 0 (normal pars oesophagea, no hyperkeratosis or lesions) to 5 (hyperkeratosis and more than 10 lesions, ulcer or occlusion of the oesophagus into the stomach) (adapted from Hessing et al*.*^45^).

### Statistical analyses

Statistical analyses were performed with the software R 4.0.3.^46^. The variables tear staining, serum NEFA concentration and hair cortisol concentration were normalized by logarithmic transformation. Areas under the recovery curves (AUC) were approximated from repeated measurements using the trapezoidal rule ${\sum }_{k=0}^{N}\left(\frac{f\left({x}_{k-1}\right)+f\left({x}_{k}\right)}{2}\right)\times \Delta xk$, where *f*(*x*) is a function and $\Delta {x}_{k}$ is the length of the *k*-th subinterval. The resilience indicator (ln(variance) based on body weight deviations was estimated using the same formulas as in the paper of Berghof et al*.*^6^.

On focal pigs only, linear mixed models were used for all variables measured once (relative growth, tear and skin lesions measured 24 h after the biopsy and before slaughter, post-mortem examinations, AUC, latencies of behaviours, total numbers of aggressive acts and posture changes during the transport challenge) with the function lmer from the R package “lme4”, except for frequencies of behaviours and scores of skin lesions and post-mortem measurements. For these variables, generalized mixed models with a Poisson distribution and Log link function were used and the function glmer from the R package “lme4”. In these models, Housing and Sex (boar versus gilt) were fixed effects, and Pen and Batch were random effects. For the repeated variables, repeated linear mixed models or repeated generalized mixed models with Housing, Sex, Time and the housing × time interaction as fixed effects, Pen and Batch as random effects, and Pig as a repeated variable were used. For hair cortisol, the Side of shaving and the Status of the animal (focal versus companion) were also included as fixed effects.

For the measurements of the heat challenge at the group level, Housing, Time period and housing × time period were used as fixed effects and Batch as a random effect. For the feed intake in the climatic respiration chambers, Housing was used as a fixed effect and Batch as a random effect.

p values below 0.05 were considered as significant effects and below 0.1 as tendencies. When a significant effect was found, pairwise comparisons between groups were made with the emmeans function of the emmeans package from R, including a Tukey correction. Data are presented as means ± SEM.

## Results

### Transport challenge

**Figure 2:**
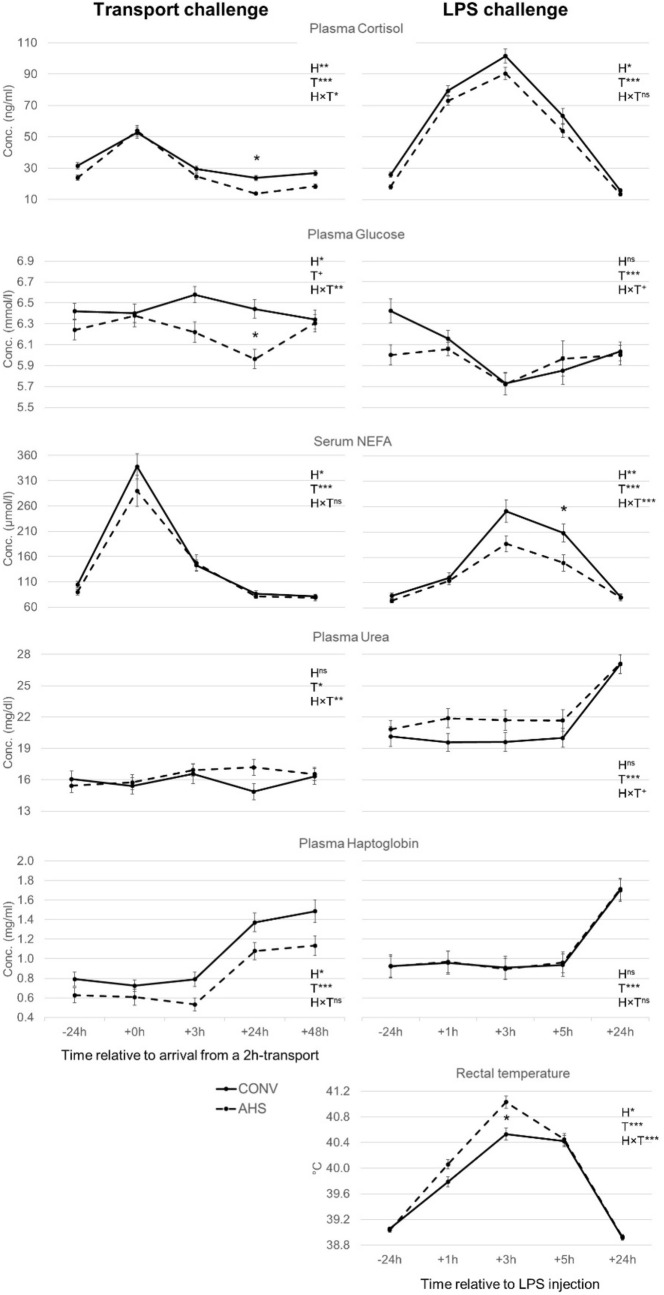
Plasma cortisol, plasma glucose, serum NEFA, plasma urea and plasma haptoglobin for both the transport and LPS challenge, and rectal temperature for the LPS challenge, of pigs housed in an alternative (AHS, dotted line) or conventional (CONV, solid line) system. *H* housing effect, *T* time effect, *HxT* housing × time interaction; ***p < 0.001; **p < 0.01, *p < 0.05, ^+^p < 0.10, ^ns^non significant. In case of interaction effects, within-timepoint differences between housing system are indicated by *. Please note, different Y-axis scaling. For the transport challenge, t = 0 h corresponds to the arrival of the pigs to the farm after the 2 h-transport.

Figure 2 shows blood parameter concentrations around transport. Plasma cortisol levels were affected by housing (p = 0.0035), time (p < 0.0001) and their interaction (p = 0.035), with lower cortisol concentrations at + 24 h in AHS compared to CONV pigs. AHS pigs also had a lower AUC of cortisol (p = 0.0010). Glucose levels were affected by housing (p = 0.0047), tended to be affected by time (p = 0.075), and were affected by the housing × time interaction (p = 0.0094). AHS pigs showed a drop in glucose at + 24 h compared to + 2 h and + 48 h, and had lower glucose concentrations at this time point than CON pigs. AUC of glucose was also lower for AHS pigs (p = 0.0023). NEFA levels increased during transport (p < 0.0001) and were higher for CONV than for AHS pigs (p = 0.047). AUC of NEFA was unaffected by housing. The course of urea levels over time (p = 0.021) was affected by housing (housing × time effect, p = 0.0088), without significant pairwise differences. The AUC of urea was unaffected by housing. Haptoglobin levels were affected by time (p < 0.0001), with increasing levels from + 3 h onwards, and were lower in AHS than in CONV pigs (p = 0.026). The AUC of haptoglobin was also lower for AHS pigs (p = 0.021).

Titers of IgM binding KLH were increased (time effect, p = 0.0003) 48 h post-challenge (5.33 ± 0.12) compared to 24 h before (5.02 ± 0.12) and 24 h after (5.07 ± 0.12) and were higher in AHS (5.42 ± 0.13) than in CONV pigs 4.86 ± 0.13, p = 0.0002). KLH-IgG titers (p < 0.0001) were higher in AHS than in CONV pigs too (AHS = 5.11 ± 0.08, CONV = 4.70 ± 0.08), without a time effect. Similarly, PC-BSA-IgM titers were higher in AHS pigs (p = 0.0081; AHS = 7.32 ± 0.13, CONV = 6.85 ± 0.13), but were unaffected by time. PC-BSA-IgG titers were unaffected by housing but showed a time effect (p = 0.011), with higher levels at + 24 h (5.50 ± 0.47) than at − 24 h (5.22 ± 0.47) and + 48 h (5.34 ± 0.47). MBP-IgM titers were higher (p = 0.0012) at 48 h post-challenge (7.27 ± 0.14) than 24 h before (6.99 ± 0.14) and 24 h post-challenge (6.84 ± 0.14) and were unaffected by housing. MBP-IgG titers were higher (p = 0.0001) + 24 h post-challenge (5.12 ± 0.25) compared to 24 h before (4.71 ± 0.25) and 48 h after (4.51 ± 0.25) and were unaffected by housing. None of the antibody titers were affected by the housing × time interaction.

**Figure 3:**
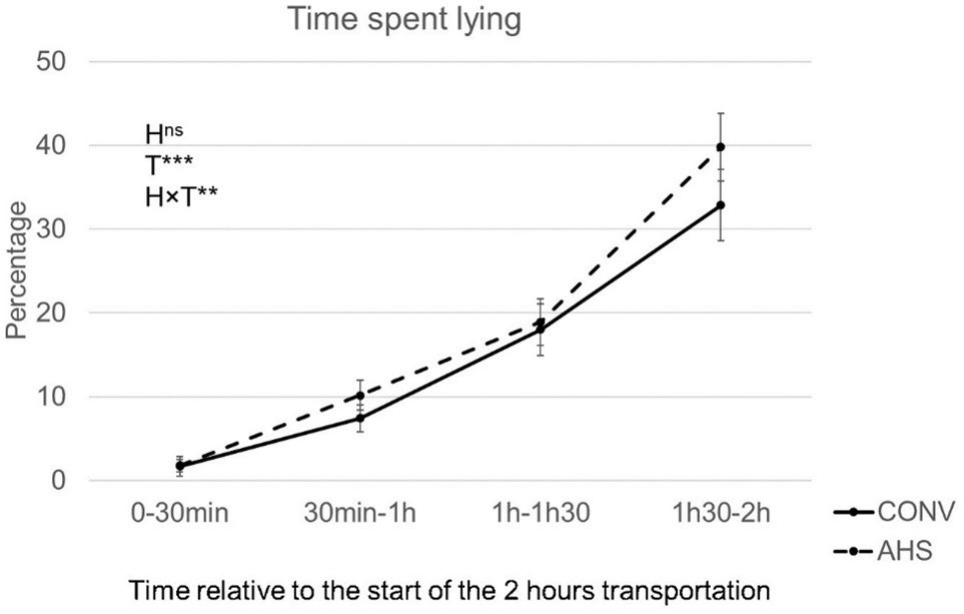
Time spent lying during the transport challenge of pigs housed in an alternative (AHS, dotted line) or conventional (CONV, solid line) system. *H* housing effect, *T* time effect, *HxT* housing × time interaction; ***p < 0.001; **p < 0.01, ^ns^non significant. In case of interaction effects, within-timepoint differences between housing system are indicated by *.

The percentage of time spent lying over time (p < 0.0001; Fig. 3) was affected by housing (housing × time effect, p = 0.0097), without significant pairwise differences. AHS pigs showed more posture changes (p = 0.011; AHS = 3.67 ± 0.11, CONV = 3.41 ± 0.11). The number of skin lesions was affected by the housing × time interaction (p = 0.012), but pairwise differences showed no differences between AHS pigs before or after the challenge, and in both housing systems lesions increased after transport (AHS: − 24 h = 2.42 ± 0.19, + 24 h = 3.65 ± 0.19; CONV: − 24 h = 2.46 ± 0.19, + 24 h = 3.52 ± 0.19). AHS pigs tended to show more aggressive interactions than CONV pigs (p = 0.083; AHS = 2.04 ± 0.23, CONV = 1.50 ± 0.23). CONV pigs gained relatively more weight during the challenge than AHS pigs (p = 0.017; AHS = 2.3% ± 0.8 of their original body weight, CONV = 3.4% ± 0.8). Tear staining did not change in response to the challenge and was not affected by housing.

### LPS challenge

Figure 2 shows blood parameter concentrations around the LPS challenge. Cortisol levels and the AUC of cortisol were lower for AHS than for CONV pigs (p = 0.030 and p = 0.038, respectively), and cortisol levels peaked at + 3 h (time effect, p < 0.0001). Glucose levels dropped at + 3 h (time effect, p < 0.0001), but both levels and AUC of glucose were unaffected by housing. NEFA levels were affected by housing (p = 0.0068), time (p < 0.0001) with a peak at + 3 h, and by the housing × time interaction (p = 0.0007) with higher levels for CONV than for AHS pigs at + 5 h. The AUC of NEFA was larger for CONV than for AHS pigs (p = 0.025). Urea was affected by time only (p < 0.0001), with a sudden increase 24 h post challenge. Haptoglobin started to increase at + 24 h (p < 0.0001), and was unaffected by housing.

Rectal temperature was affected by housing (p = 0.034), time (p < 0.0001) and their interaction (p < 0.0001). Temperatures increased till + 3 h before going back to baseline levels at + 24 h. At + 3 h, AHS pigs had higher temperatures than CONV pigs.

KLH-IgM was affected by housing (p < 0.0001) and the housing × time interaction (p = 0.027) with higher titers for AHS pigs than CONV pigs both at − 24 h (AHS = 6.34 ± 0.1, CONV = 5.78 ± 0.1) and at + 24 h (AHS = 6.38 ± 0.10, CONV = 5.64 ± 0.10) and a decrease after injection for CONV pigs only. Titers of KLH-IgG were higher in AHS (5.70 ± 0.17) than in CONV pigs (5.32 ± 0.17, p = 0.028) and were also affected by time (p = 0.016) with increasing values post-challenge (− 24 h = 5.46 ± 0.15, + 24 h = 5.56 ± 0.15). PC-BSA-IgM was affected by the housing × time interaction (p = 0.014), without significant pairwise differences (− 24 h, AHS = 7.71 ± 0.14, CONV = 7.62 ± 0.14; + 24 h, AHS = 7.88 ± 0.14, CONV = 7.51 ± 0.14). PC-BSA-IgG increased over time (p = 0.0049, − 24 h = 5.78 ± 0.47, + 24 h = 5.89 ± 0.47), but was unaffected by housing or its interaction with time. Time also affected titers of MBP-IgM (p = 0.023, − 24 h = 7.53 ± 0.22, + 24 h = 7.76 ± 0.22) and MBP-IgG (p = 0.005, − 24 h = 4.63 ± 0.13, + 24 h = 4.47 ± 0.13), but housing did not.

Skin lesions (AHS = 2.44 ± 0.89, CONV = 2.54 ± 0.88) and tear staining (AHS = 0.68 ± 0.094, CONV = 0.62 ± 0.057) did not change in response to the challenge, and were unaffected by housing. Also, relative weight change was unaffected by housing (AHS = 0.99 ± 0.40%, CONV = 0.66 ± 0.40%) (data not shown).

### Heat stress challenge

**Figure 4:**
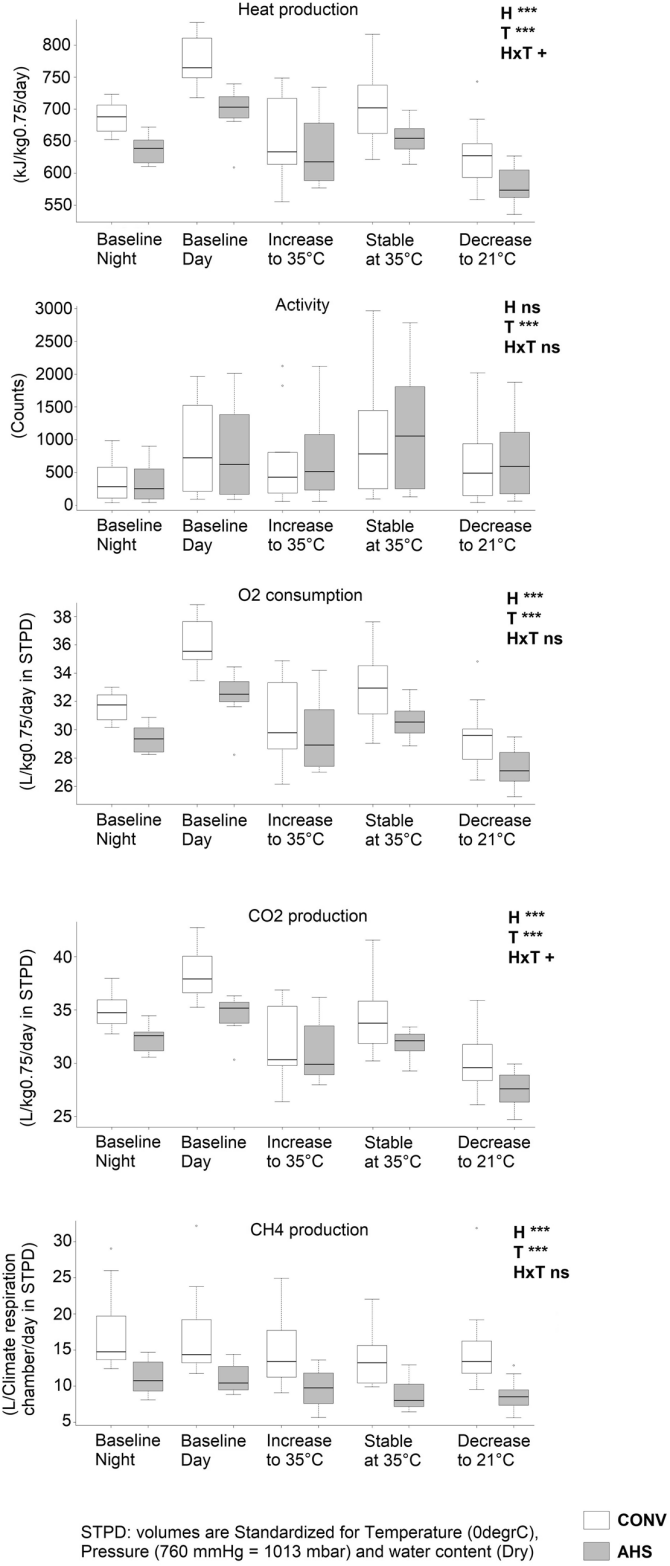
Heat production, O_2_ consumption, CO_2_ production, CH_4_ production and activity during the heat stress challenge of pigs housed in an alternative (AHS, in grey) or conventional system (CONV, in white). *H* housing effect, *T* time effect, *HxT* housing × time interaction; ***p < 0.001; + p < 0.10; ^ns^non significant.

Figure 4 shows the O_2_ consumption, CO_2_, CH_4_ and heat production and activity as assessed in the climatic respiration chambers per time period of interest. A more detailed graph is shown in Supplementary Fig. 1. All variables were affected by time period (p < 0.0001). Baseline O_2_ consumption and CO_2_ and heat production were lower during the night than during the day. During the 2 h heating up of the chambers, levels of these variables went down, as compared to baseline day levels. The subsequent 2 h period at 35 °C showed an increase in O_2_ consumption and CO_2_ and heat production again, but levels did not reach baseline day levels. During the 2 h cooling down of the chamber, pigs showed the lowest levels of O_2_ consumption and CO_2_ and heat production. Methane production was lower during the heating up, the 2 h 35 °C period and the cooling down than during the baseline levels of the day and the night (which did not differ from each other).

CONV pigs produced more heat (p < 0.0001), more O_2_ (p < 0.0001), CO_2_ (p = 0.00013) and CH_4_ (p < 0.0001), irrespective of time period, as there were no housing × time interactions, and housing effects were already found at baseline.

The baseline activity was lower at night than at day. Compared to baseline day levels, activity was lower during the heating up, increased during the 35 °C period and decreased again during the cooling down. Activity levels were not affected by housing.

**Figure 5:**
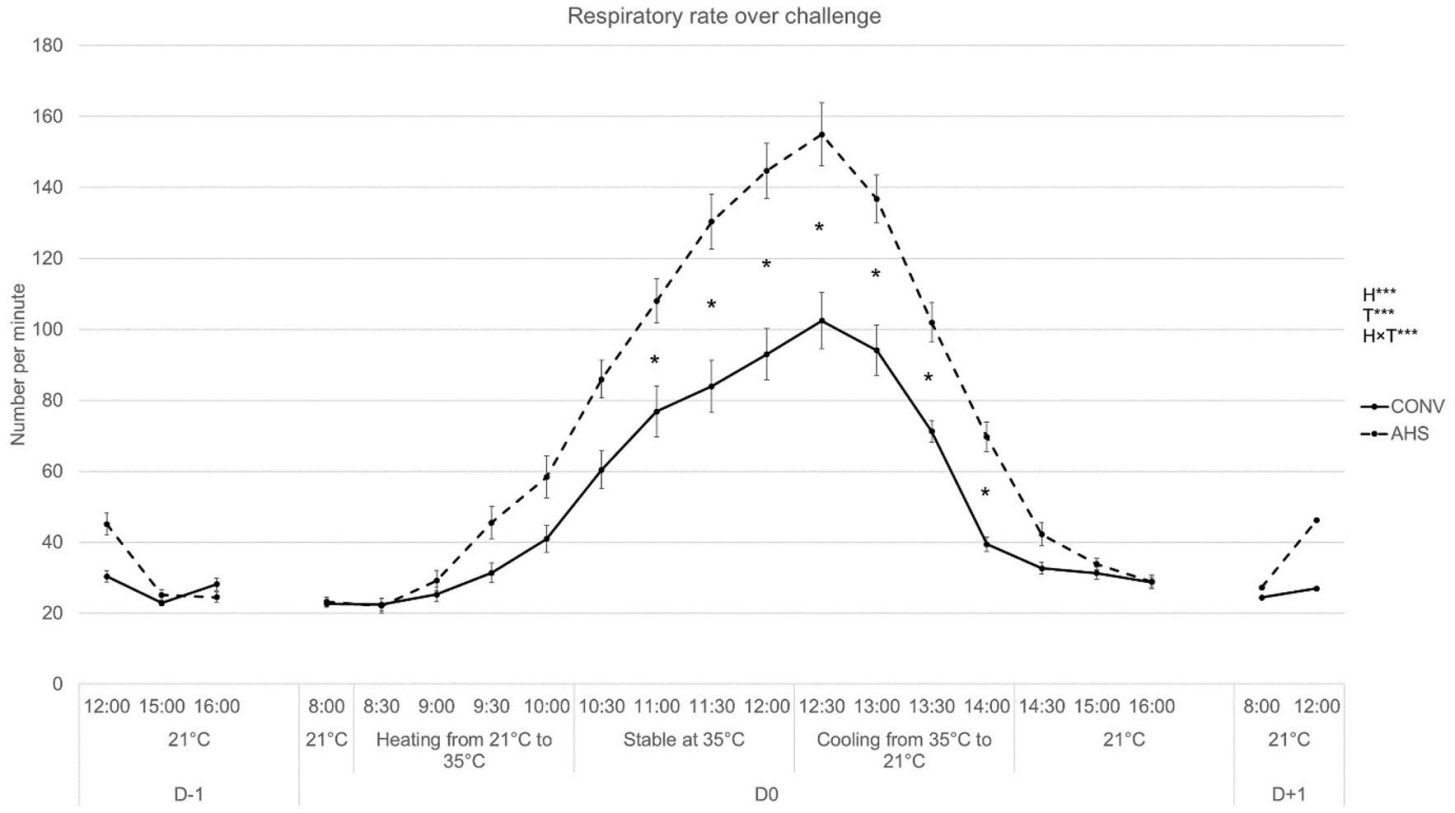
Respiratory rate during the heat stress challenge of pigs housed in an alternative (AHS, dotted line) or conventional (CONV, solid line) system. *H* housing effect, *T* time effect, *HxT* housing × time interaction; ***p < 0.001; *p < 0.05. In case of interaction effects, within-timepoint differences between housing system are indicated by *.

Respiratory rate (RR) and the related AUC as measured at the individual level were affected by housing (p < 0.0001 and p = 0.00081, respectively), by time (p < 0.0001) and their interaction (p < 0.0001). From 30 min after the 35 °C was reached till 1h30 after the cooling process was started post-challenge, AHS pigs had a higher RR than CONV pigs (Fig. 5).

CONV pigs gained relatively more weight during the challenge period than AHS pigs (p = 0.043; AHS = 0.58% ± 0.29 of their pre-challenge body weight, CONV = 1.41% ± 0.29). Effect of housing on feed intake (AHS 13.5 ± 0.44 kg; CONV 14.4 ± 0.44 kg) did not reach significance (p = 0.058). Skin lesions were higher post-challenge than pre-challenge (p = 0.0031; − 24 h = 2.78 ± 0.050 and + 24 h = 2.88 ± 0.050), suggesting an increase in the number of aggressive events. The heat stress challenge caused an increase of the tear staining area (p = 0.029; − 24 h = 0.56 ± 0.12 arb. unit, + 24 h = 0.79 ± 0.055 arb. unit).

### Wound healing challenge

**Figure 6:**
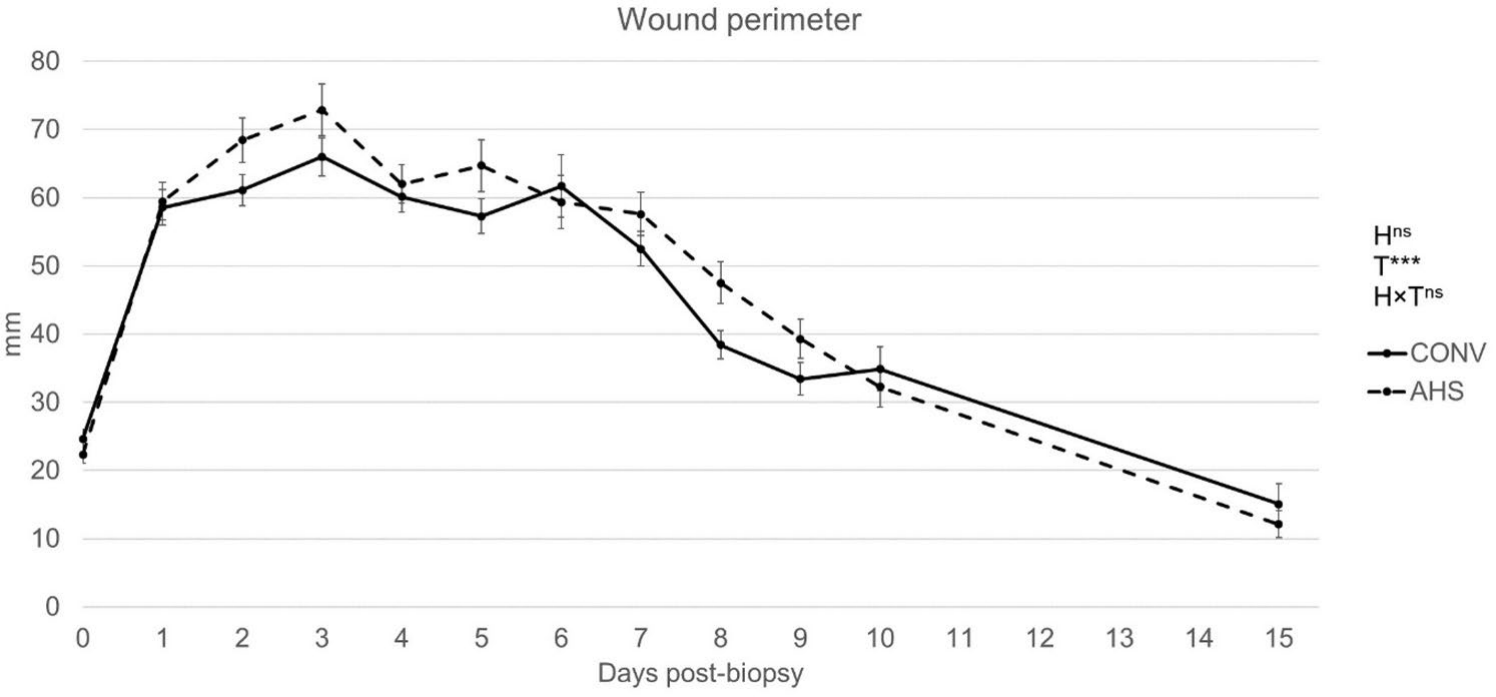
Wound perimeter as a reflection of the wound healing process of pigs housed in an alternative (AHS, dotted line) or conventional (CONV, solid line) system. *H* housing effect, *T* time effect, *HxT* housing × time interaction; ***p < 0.001; ^ns^non significant.

The healing perimeter of the biopsy was affected by time (p < 0.0001), with an increase up to 3 days post-challenge followed by a progressive decrease over time (Fig. 6), but was unaffected by housing. AHS pigs presented more skin lesions on the day of the challenge (p = 0.0012; AHS = 2.96 ± 0.17, CONV = 2.53 ± 0.16). Tear staining measured right before wound healing challenge, possibly indicating the stress experienced the previous days, was higher for CONV pigs compared to AHS pigs.

### Growth, accumulation of cortisol in hairs, and post-mortem assessment

**Figure 7:**
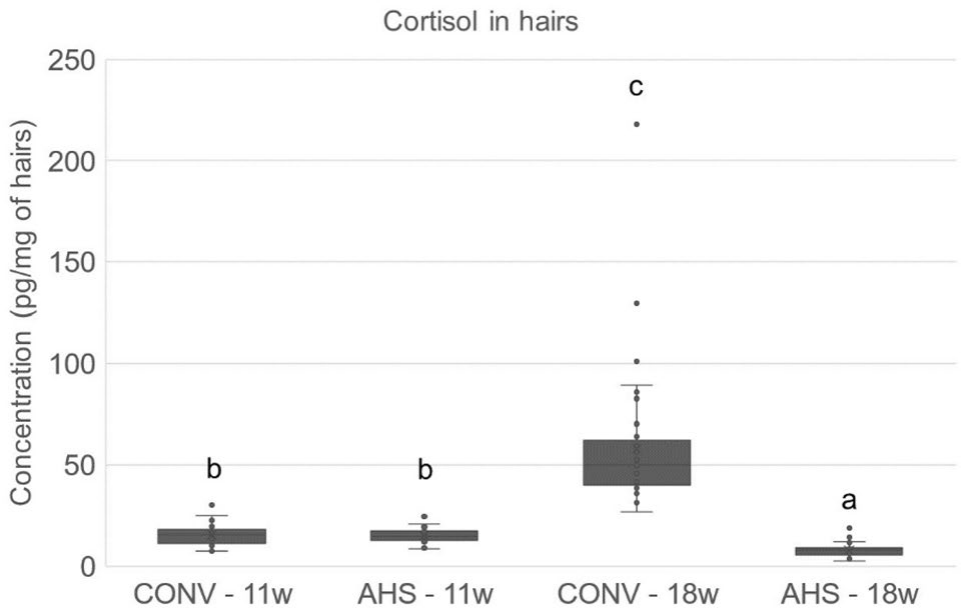
Cortisol concentration in hairs measured before (11 weeks of age) and at the end (18 weeks of age) of the period of challenges in pigs housed in an alternative (AHS) or conventional (CONV) system. Letters were attributed for significantly different values a < b < c; p < 0.0001.

Body weight gain over time (p < 0.0001) was not affected by housing. However, the Ln(variance) of the weights of CONV pigs was higher than that of AHS pigs (p = 0.018; AHS = − 1.90 ± 0.22 arb. unit, CONV = − 1.58 ± 0.22 arb. unit). Tear staining was affected by time only (p < 0.0001) with lower values at 11 and 12 weeks than at the weeks thereafter. The concentration of cortisol in hairs was affected by time (p < 0.0001), housing (p < 0.0001) and their interaction (p < 0.0001). At 11 weeks of age, hair cortisol levels were similar for CONV and AHS pigs. However, in CONV pigs, cortisol concentration in hairs increased profoundly between 11 and 18 weeks of age while in AHS pigs it decreased over time, resulting in a treatment difference at 18 weeks of age (Fig. 7). The side of shaving at 18 weeks of age and the status of the animal (focal versus companion) had no effects on the cortisol concentration in hairs. At slaughter, AHS pigs had more skin lesions (p = 0.014; AHS = 2.83 ± 0.17, CONV = 2.45 ± 0.17). Pericarditis (only one CONV pig affected) and pleurisy (9 CONV pigs and 4 AHS pigs had a score of 1) hardly occurred. Scores of pneumonia (17.4 ± 1.1) and gastric lesions (2.92 ± 0.13) were not affected by housing.

### Sex effects

Only the significant sex effects are described below and summarized in Table 1. Boars had higher levels and AUC of glucose than gilts in both the transport and LPS challenge, and lower levels and AUC of urea levels. Rectal temperature during the LPS challenge was higher for gilts. Boars changed their posture more often, showed more aggressive acts, and presented more skin lesions during transport. Skin lesions measured at the LPS, heat stress and wound challenge were also higher for boars. During the heat challenge, boars had a higher RR than gilts as well as higher relative weight gain. Around the wound challenge, gilts recovered slower than boars, but they had a smaller tear staining area. At slaughter, the pneumonia score was higher for boars compared to gilts.

**Table 1:** Significant effects of sex (Boar versus Gilt) on variables measured during various challenges (lsmeans ± SEM).

Challenge	Variable	Boar	Gilt	p value
2 h-Transport	Plasma glucose (mg/100 ml)	116 ± 2.3	112 ± 2.3	0.0020
	Plasma urea (mmol/l)	2.38 ± 0.2	3.00 ± 0.2	< 0.0001
	Aggression (n)	2.60 ± 0.43	1.45 ± 0.43	< 0.0001
	Posture changes	3.66 ± 0.1	3.42 ± 0.1	< 0.0001
	Skin lesions (n)	3.19 ± 0.19	2.84 ± 0.19	< 0.0001
LPS injection	Plasma glucose (mg/100 ml)	110 ± 1.9	106 ± 1.9	0.022
	Plasma urea (mmol/l)	3.18 ± 0.12	4.15 ± 0.12	< 0.0001
	Rectal temperature (°C)	39.8 ± 0.05	39.9 ± 0.05	0.039
	Skin lesions (n)	2.60 ± .080	2.38 ± 0.081	0.0095
Heat stress	Respiratory rate (n/min)	59.0 ± 2.6	52.1 ± 2.6	0.0017
	Relative growth (%)	1.45 ± 0.25	0.54 ± 0.25	0.00067
	Skin lesions (n)	2.95 ± 0.065	2.71 ± 0.066	0.0091
Wound healing	Healing perimeter^a^ (mm)	46.4 ± 3.4	50.7 ± 3.4	0.0058
	Skin lesions (n)	2.80 ± 0.14	2.69 ± 0.14	0.020
	Tear staining (arb. unit)	0.79 ± 0.14	0.42 ± 0.07	0.026
Dissection	Pneumonia score (arb. unit)	3.49 ± 0.61	3.41 ± 0.61	0.028

^a^Healing perimeter: perimeter of the wound caused by biopsy.

## Discussion

In this study, resilience was assessed by subjecting pigs to a series of challenges. Pigs from the alternative housing system (AHS) were expected to have an improved resilience compared to pigs from conventional housing (CONV). This hypothesis was based on the AHS promoting socialization, providing a more gradual and prolonged weaning transition, and ample provision of environmental enrichment and space in the AHS, to support the expression of important species-specific behaviours. This putative improved resilience would then be demonstrated in a faster recovery from the challenges, and lower expression of indicators reflecting poor welfare and “wear and tear” on the body over the complete experimental period.

As hypothesized, AHS pigs showed a faster recovery and/or a lower response than CONV pigs in several physiological indicators around the 2 h-transport (cortisol, glucose and non-esterified fatty acids, NEFA), and the LPS challenge (cortisol and NEFA). Only in the AHS pigs, the elevation of corticosteroid production following stressful conditions caused a drop in glucose, which will turn the body into a catabolic state, activating the use of alternative fuels such as NEFA^47^. Transport is one of the most well-known stressors in pig production, involving handling, loading, and packing in a truck^48^, and usually leading to a rise in cortisol^49–51^. A less pronounced cortisol response to transport in AHS pigs is in line with a previous study in which pigs kept in enriched and larger pens showed a lower increase after transport^52^ or no increase at all^53^. Similar to other studies, transport also increased the levels of haptoglobin, an acute phase protein^49–51^. This increase could both reflect acute stress^54^, or, alternatively, skin inflammation caused by the lesions induced during fighting at transport. The latter is less likely, however, as deep, large or bleeding wounds were never observed. Although the dynamics of the haptoglobin response did not differ between housing systems, AHS pigs showed overall lower levels of haptoglobin than CONV pigs, which corresponds with other studies on enriched housed pigs^43,55,56^.

The LPS challenge induces a sickness response in pigs, which is associated with increased cortisol levels, a fever response, decreased activity, and reduced feed and water intake^57–59^, mimicking the response to infection. Although AHS pigs showed a less pronounced response and/or faster recovery in cortisol and NEFA, their peak in rectal temperature at 3 h post LPS injection was higher, with a similar area under the curve. Developing fever may be an efficient strategy to fight infection by stimulating proliferation of immune cells and limiting micro-organism proliferation^60^. A number of studies suggest indeed that enriched housed pigs more efficiently combat infections. Pigs provided with larger living space had lower lung lesions induced by *M. hyopneumonia*^61^. Similarly, pigs housed in enriched social and environmental conditions demonstrated a faster viral clearance compared to pigs raised in barren pens, and less lung lesions^28^ when exposed to a porcine reproductive and respiratory virus—*Actinobacillus pleuropneumoniae* co-infection. Disease challenges can generate pain and general fatigue, compromising animal welfare^62,63^. Moreover, it has recently been shown that induction of a sickness response (by LPS) was a risk factor for the expression of damaging behaviours like tail biting^64^. Tail biting is a common problem in pig farming leading to impaired welfare and health, as well as production loss. Taken together, the more favourable response to the LPS challenge with quicker recovery in the AHS pigs may thus have important welfare consequences.

We expected a faster wound healing in AHS pigs than in CONV pigs. However, no effects of housing system were found, while a better wound healing has been demonstrated for pigs receiving cognitive enrichment^65^.

Heat stress is metabolically demanding for pigs, affecting carbohydrate and lipid metabolism, e.g. reflected in an increased NEFA concentration and respiratory rate^66^. AHS and CONV pigs differed in responses to the heat challenge, as AHS pigs showed a lower growth rate and tended to eat less while being in the climate respiration chambers. During heat stress periods, reducing feed intake has been shown to be a good strategy to minimize metabolic heat production^67^. It cannot be excluded, though, that the lower feed intake and weight gain in AHS pigs were related to the larger difference in environmental quality between the climate respiration chambers and home pens for those pigs compared to the CONV pigs, due to the lack of bedding and extra toys. Switching from enriched to barren environments resulted in frustration, apathy and more aggressive interactions in previous studies^68,69^. During the challenge, AHS pigs showed a higher respiratory rate than CONV pigs. It might be that the CONV pigs developed a different thermoregulatory strategy during the heat wave. Even though not scored systematically, more manure and urine was seen on the floor of the metabolic chambers with CONV pigs, which were possibly used to wallow in, as observed in other studies^70,71^. During the heat stress, pigs were expected to increase heat production and consume more O_2_ due to the increase of respiratory rate trying to dissipate the extra heat^72^. In this study, however, O_2_ consumption was unchanged during the heat wave but increased post-entrance to the chamber and right after the heat wave, which was unrelated to an increased general activity. Those two peaks might reflect a delayed response or recuperation to stressful events: the transport to the metabolic chamber on one side, and the heat stress on the other side. As expected, CH_4_ production decreased during the heat wave, probably due to a reduction in feed intake^72,73^. Activity followed an expected pattern with lower intensity during the night compared to the day. However, the highest intensity was observed when temperature was 35 °C, which might be related to frequent changing of the lying postures from belly to the side due to the pigs’ discomfort. Both activity and heat production showed a peak right after entrance to the chambers, probably related to exploration of the new environment, and right after the heat wave, which might be due to compensatory feed intake. Heat production was higher at night, during the day and when temperature was at 35 °C for CONV pigs compared to AHS pigs. Overall, the CONV pigs also consumed more O_2_ and produced more CH_4_ and CO_2_ during the day. These differences between CONV and AHS pigs might point to a higher metabolic rate in CONV pigs. This could be in line with the study from Matuszewich and Yamamoto^74^ and De Jong et al.^75^, who demonstrated hyperthermia in rats and pigs, respectively, and suggested that this was a reflection of long-lasting effects of chronic stress. An increased standard metabolic rate, determined by O_2_ consumption, was also observed in brown trout following a social stressor^76^. More research is needed on a potential difference in metabolic rate in pig in diverging housing conditions, as this would also be relevant to sustainability of pig farming.

Stressors, via their effects on the hypothalamic–pituitary–adrenal axis and sympathetic autonomous nervous system, affect the immune system^77^. Natural antibodies (NAb) are important as a first line of defence against pathogens due to their poly-specificity^78^, and part of them, natural auto-antibodies, also bind to self-antigens. It has been shown that infection^40^, but also acute stressors, such as regrouping^55^ and transport^40^, can change NAb levels in pigs, including those binding KLH, MBP and phosphorylcholine (PC). In our study, levels of several natural (auto)antibodies changed after transport and LPS injection. Unlike a previous study, these changes were generally not depending on housing system, except for IgM binding KLH after the LPS challenge, which increased more in AHS pigs. However, irrespective of time of sampling, AHS pigs showed higher levels of IgM binding PC, a cell wall component which is exposed after cell damage and inflammation, around the transport challenge, and higher KLH-IgM and KLH-IgG levels at both the transport and LPS challenge. Also in another study, enriched housed pigs showed higher levels of antibodies binding KLH^55^, although others found the opposite^28,79^. KLH is a large glycoprotein with different epitopes recognized by heterogeneous NAbs. Increased KLH NAb levels might reflect better resilience, as favourable associations between KLH NAbs and disease resistance and survival have been demonstrated in chickens^6,80–82^. Similarly, in pigs, a recent paper reported that pigs reaching market age (a proxy for survival) had higher IgG NAb binding KLH^83^. However, little is known about the benefits or drawbacks to have high levels of antibodies and the conclusions need to be taken with caution.

It was expected that a better resilience to the challenges would also be reflected in indicators of “wear and tear” on the body over the complete experimental period. Indeed, AHS pigs had lower cortisol accumulation in hairs from 11 to 18 weeks of age, i.e. over the weeks in which they were exposed to challenges, than CONV pigs, reflecting lower accumulative stress. This could partly be due to the chronic stress of suboptimal housing in CONV pigs, as lack of environmental enrichment induces changes in the HPA-axis^53^. CONV pigs also presented larger tear staining areas than AHS pigs when measured around 18 weeks of age. Tear staining, which may reflect chronic stress and therefore a poorer welfare^42,84^. Lastly, in spite of the fact that they gained less weight during the transport and heat stress challenge, AHS pigs had a lower variance of their weight development over time. The lower weight gain around the transport challenge might be related to a higher general activity, with more posture changes and aggressive interactions. Regarding the heat stress challenge, the decrease of feed intake, as an adaptive strategy to reduce heat production, might explain the lower weight gain at this period. In other studies, a lower variance of their weight development has been associated with improved resilience^6^. Thus, this overall indicator would be in accordance with all the other ones and might indicate a higher resilience in the AHS pigs. It should be noted, though, that this did not lead to a difference in damage to lungs and stomach as assessed post-mortem.

Contrary to expectations, AHS pigs had a higher number of skin lesions when measured before the wound challenge and before slaughter, which may reflect more aggressive interactions, even though the overall number of skin lesions was low. Increased aggressiveness was also noticed for pigs enriched pre-weaning compared to barren ones at weaning and mixing^69^. However, in the same study, the authors found an opposite result when the enrichment was delivered post-weaning. Other studies showed a reduction of aggression post-weaning when piglets received pre-weaning enrichment and early mingling^13,85,86^. In the current study, piglets were mixed in a bigger group during transport than what they were used to and had to adapt to transportation. This specific context makes the comparison with existing studies difficult.

As expected, boars showed more aggression^87^ which is likely related to their concentrations of sexual steroids^88^. The current study also exposed differences between males and females regarding resilience. Boars showed increased levels of glucose, while gilts had higher urea concentrations, reflecting more protein metabolism, in both the transport and LPS challenge. This indicates that both sexes differ in their metabolic response to stress. Gilts also had higher rectal temperatures during the LPS challenge, and presented a slower wound healing. At the end of the experiment, they had less tear staining and lower pneumonia scores. Those differences might reveal different strategies to fight against challenges. Others also found that females and males seem to react differently to stress depending on the nature of the stressor, its duration, the age of the individual and the behavioural or physiological phenotype considered^89,90^. More research is needed on sex effects on resilience.

## Conclusion

Pigs raised and kept in an alternative housing system as compared with those in a conventional system showed better physiological recoveries from challenges, as well as less signs of long-term “wear and tear” on the body. Providing social and environmental enrichment that promotes socialization, the expression of species-specific behaviours and, overall, the satisfaction of their essential needs appears to be a promising approach to favour resilience in pigs. Further research is needed to confirm enhanced resilience when welfare is promoted via increased opportunities to show natural behaviour.

## Supplementary Information

Supplementary Information.
