# Supplementary material for: A multi-suckling system combined with an enriched housing environment during the growing period promotes resilience to various challenges in pigs
Source: Sci Rep. 2022 Apr 26;12:6804. doi: 10.1038/s41598-022-10745-4 (PMC9043182; doi:10.1038/s41598-022-10745-4)
Supplement: Supplementary file 1 — Supplementary Information. [file 41598_2022_10745_MOESM1_ESM.docx]

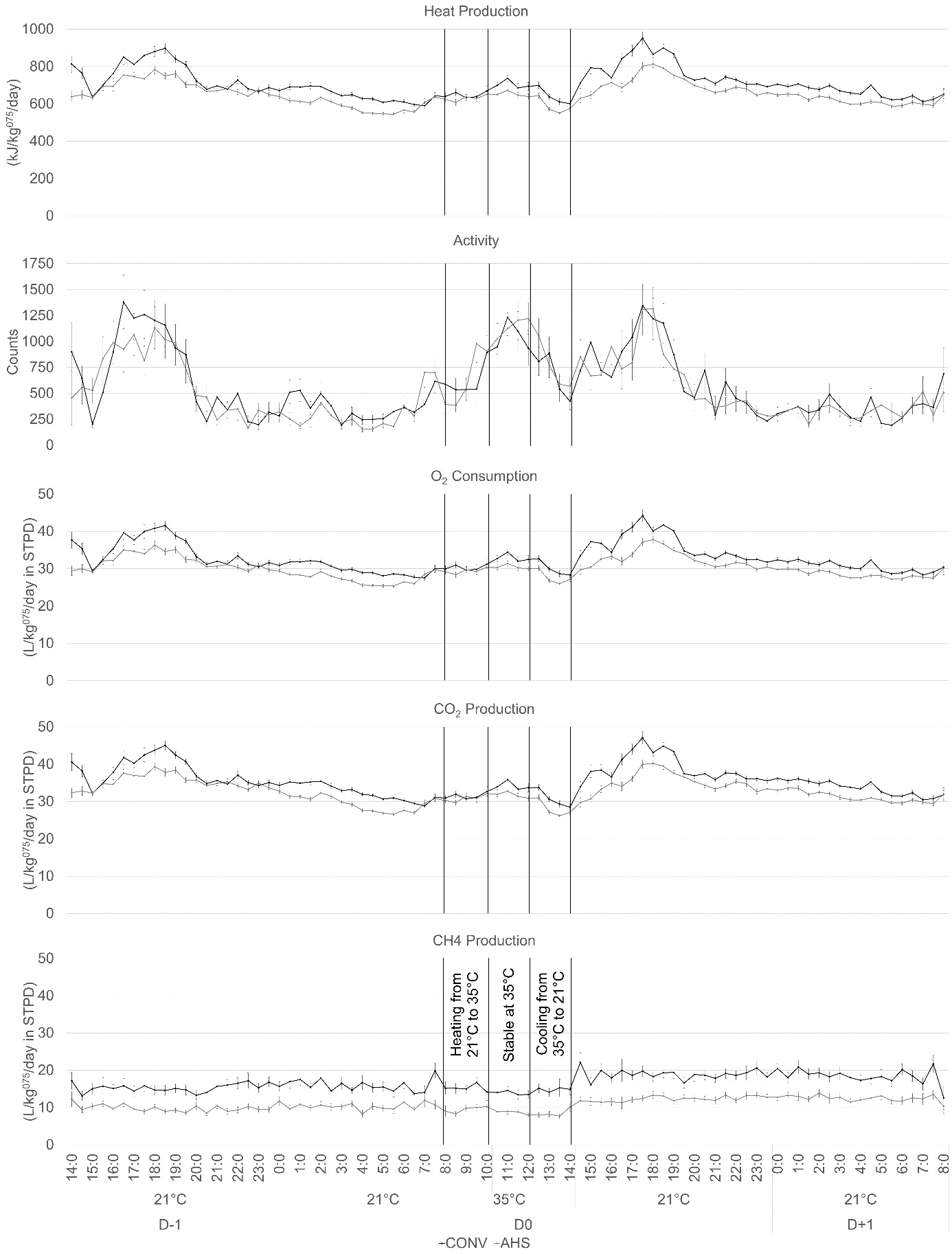


**Supplementary Figure 1: Heat production, O_2_ consumption, CO_2_ production, respiratory quotient, CH_4_ production and activity during the heat stress challenge of pigs housed in an alternative (AHS, grey line) or conventional system (CONV, black line).**
